# Current Status of Biparametric MRI in Prostate Cancer Diagnosis: Literature Analysis

**DOI:** 10.3390/life12060804

**Published:** 2022-05-28

**Authors:** Mason James Belue, Enis Cagatay Yilmaz, Asha Daryanani, Baris Turkbey

**Affiliations:** Molecular Imaging Branch, National Cancer Institute (NCI), National Institutes of Health (NIH), Bethesda, MD 20892-9760, USA; mason.belue@nih.gov (M.J.B.); enis.yilmaz@nih.gov (E.C.Y.); asha.daryanani@nih.gov (A.D.)

**Keywords:** prostate cancer, bpMRI, mpMRI, DCE MRI

## Abstract

The role of multiparametric MRI (mpMRI) in the detection of prostate cancer is well-established. Based on the limited role of dynamic contrast enhancement (DCE) in PI-RADS v2.1, the risk of potential side effects, and the increased cost and time, there has been an increase in studies advocating for the omission of DCE from MRI assessments. Per PI-RADS v2.1, DCE is indicated in the assessment of PI-RADS 3 lesions in the peripheral zone, with its most pronounced effect when T2WI and DWI are of insufficient quality. The aim of this study was to evaluate the methodology and reporting in the literature from the past 5 years regarding the use of DCE in prostate MRI, especially with respect to the indications for DCE as stated in PI-RADS v2.1, and to describe the different approaches used across the studies. We searched for studies investigating the use of bpMRI and/or mpMRI in the detection of clinically significant prostate cancer between January 2017 and April 2022 in the PubMed, Web of Science, and Google Scholar databases. Through the search process, a total of 269 studies were gathered and 41 remained after abstract and full-text screening. The following information was extracted from the eligible studies: general clinical and technical characteristics of the studies, the number of PI-RADS 3 lesions, different definitions of clinically significant prostate cancer (csPCa), biopsy thresholds, reference standard methods, and number and experience of readers. Forty-one studies were included in the study. Only 51% (21/41) of studies reported the prevalence of csPCa in their equivocal lesion (PI-RADS category 3 lesions) subgroups. Of the included studies, none (0/41) performed a stratified sub-analysis of the DCE benefit versus MRI quality and 46% (19/41) made explicit statements about removing MRI scans based on a range of factors including motion, noise, and image artifacts. Furthermore, the number of studies investigating the role of DCE using readers with varying experience was relatively low. This review demonstrates that a high proportion of the studies investigating whether bpMRI can replace mpMRI did not transparently report information inherent to their study design concerning the key indications of DCE, such as the number of clinically insignificant/significant PI-RADS 3 lesions, nor did they provide any sub-analyses to test image quality, with some removing bad quality MRI scans altogether, or reader-experience-dependency indications for DCE. For the studies that reported on most of the DCE indications, their conclusions about the utility of DCE were heavily definition-dependent (with varying definitions of csPCa and of the PI-RADS category biopsy significance threshold). Reporting the information inherent to the study design and related to the specific indications for DCE as stated in PI-RADS v2.1 is needed to determine whether DCE is helpful or not. With most of the recent literature being retrospective and not including the data related to DCE indications in particular, the ongoing dispute between bpMRI and mpMRI is likely to linger.

## 1. Introduction

Prostate cancer (PCa) is the second-leading cause of cancer-related deaths among individuals born biologically male [1]. Multi-parametric MRI (mpMRI) has been recognized as an important tool in the detection, localization, staging, and management of prostate cancer [2]. mpMRI demonstrates high sensitivity and specificity for identifying clinically significant prostate cancer (csPCa), with cancer detection rates up to 80–90% [3,4,5]. Depending on the patient selection, mpMRI has also demonstrated a negative predictive value (NPV) of 63–98% and could reduce unnecessary biopsies by more than 27% [6,7]. The high incidence of PCa in addition to the strength of mpMRI necessitates widespread adoption of prostate MRI. As a response to mpMRI’s growth, the Prostate Imaging Reporting and Data System (PI-RADS) guidelines were introduced in 2012 as PI-RADS v1 to standardize prostate mpMRI acquisition, interpretation, and reporting [8].

### 1.1. Role of I.V. Contrast (DCE Imaging) in Prostate Cancer Imaging and Controversies

Since the genesis of PI-RADS, there have been updates and refinements, with PI-RADS v2.0 being released in 2015 and the latest guidelines being PI-RADS v2.1, introduced in 2019 [9]. The updates/refinements, introduced in response to observed inter-/intrareader variability in PI-RADS v1, include changing the roles/responsibilities of the various mpMRI sequences (T2WI, DWI, ADC, DCE). One of the many important recommendations in PI-RADS v2.1 details the uses of anatomical T2-weighted imaging combined with functional dynamic contrast enhancement (DCE) and diffusion-weighted imaging (DWI), with DCE producing the most controversy. The debate surrounding I.V. contrast in prostate imaging is centered on the utility of DCE imaging. The concept of dominant sequences remained unchanged in the latest PI-RADS v2.1 update, with roles for DWI in the peripheral zone and T2WI in the transition zone [10]. The role of DCE in PI-RADS v2.1 is to serve as a modifier to upgrade peripheral zone lesions from PI-RADS 3 to category 4 in the presence of early focal enhancement [6,9]. It was also mentioned in PI-RADS v2.1 that when T2WI and DWI are of insufficient diagnostic quality, DCE utilizing I.V. contrast can assist in determining the PI-RADS assessment category [10,11]. In summary, the PI-RADS v2.1 indications for DCE, and thus for I.V. contrast, are: (1) identifying PI-RADS 3 lesions that include clinically significant prostate cancer; (2) assisting in the readout of MRIs with suboptimal diagnostic quality for T2WI and DWI sequences resulting from noise/artifacts; and (3) assisting radiologists with relatively low experience in reading prostate MRIs.

The role of DCE in detecting csPCa has been a continuing controversy due to it being more time-consuming, having potentially increased risks associated with gadolinium-based contrast agents, and having increased costs, as well as the apparently minor contribution/role of DCE defined by the PI-RADS v2.1 guidelines [12,13,14]. Biparametric MRI (bpMRI) has been proposed to alleviate the limiting factors/controversies of mpMRI by omitting DCE from the examination. A major caveat for the use of bpMRI is that it does not apply to the settings involving tumor recurrence after radiation therapy, focal therapy, or radical prostatectomy. In these scenarios, DCE plays a more dominant role, since contrast enhancement is one of the most reliable features of a disease in the context of therapy-induced prostatic changes, which make PI-RADS inapplicable [15]. Another caveat related to the indications [2,3] for the use of bpMRI is the variability in diagnostic accuracy, which is significantly influenced by the experience of the readers and the quality of the MRI scan. It is claimed that less-experienced readers benefit more from DCE, with one study highlighting the more robust nature of DCE in meeting diagnostic quality, with T2WI and DWI combined only meeting the diagnostic threshold in 60% of cases compared to DCE in 93% [16]. It has been suggested that the more robust nature of DCE can be explained by the higher spatial resolution and it being less prone to motion or susceptibility artifacts. One review including 77 articles showed that reading experience and biopsy experience were the main factors that influenced diagnostic accuracy. They found that the use of bpMRI appears to be most effective with experienced readers and when good image quality is available, but DCE MRI should be used as a backup for those with less experience [17]. In summary, bpMRI has been shown to suffer in non-expert, low-volume, lower-field-strength scanners, suggesting further prospective studies be performed to specifically test these indications for DCE [18]. Owing to the heterogeneity of the studies and complexity of the disease, the impact of removing DCE on diagnostic accuracy remains to be determined.

### 1.2. Prior Reviews Comparing mpMRI against bpMRI (without DCE)

Previously published literature reviews and meta-analyses comparing pre-biopsy mpMRI versus bpMRI head-to-head have provided variable conclusions. Most studies investigating whether bpMRI can replace mpMRI directly compare the clinically significant cancer detection rates, most often by retrospectively removing DCE from mpMRI assessment. Notably, these reviews pooled studies for patients with all PI-RADS lesions ranging from category 1–5 but did not provide any insight into individual pooled PI-RADS 3 patients/lesions or any sub-analyses of scan quality or reader experience, which are the primary indications for DCE in PI-RADS v2.1. One systematic review and meta-analysis by Woo et al. included 20 studies (from January 2008–2017) and found that the performance of bpMRI was similar to that of mpMRI in the diagnosis of prostate cancer [2]. Another meta-analysis by Niu et al. included 33 studies (from January 2000–July 2017) and found that mpMRI had better pooled sensitivity (mpMRI; 0.85, bpMRI; 0.80) but similar pooled specificity (mpMRI; 0.77, bpMRI; 0.80) [19]. A meta-analysis of a similar size by Alabousi et al. included 31 studies (from January 2012–2018) and found no significant differences in the pooled sensitivity (mpMRI: 0.86, bpMRI: 0.90) or specificity (mpMRI: 0.73, bpMRI: 0.70) [20]. A larger meta-analysis by Bass et al. included 44 studies (from January 2017–June 2019) and the meta-regression revealed no differences in the pooled diagnostic estimates between bpMRI and mpMRI. The Bass et al. review was very similar to the Niu et al. and Alabousi et al. reviews, except this study added seven more papers to the final pooling, with Bass et al. mentioning that the heterogeneity of the data did not allow definitive recommendations to be made [21]. A meta-analysis by Liang et al. of 45 studies (from 2007–2019) found a slightly significant difference in sensitivity (mpMRI: 0.84, bpMRI: 0.77) but no significant difference in specificity (mpMRI: 0.82, bpMRI: 0.81) [22]. A special note should be added that most of these reviews, except for that by Bass et al., pooled studies using different PI-RADS versions because of the years the studies were published. Since the roles of the various sequences (T2, DWI, ADC, and DCE) changed between versions and evidence of inter-/intrareader variability has been shown to exist between the PI-RADS versions, studies from different PI-RADS eras are more difficult to pool and directly compare.

Only a handful of prior reviews gave PI-RADS 3 lesion-specific advice. Of these, a smaller review of ten studies that found no significant differences in sensitivity or specificity defended the use of biopsy for all PI-RADS 3 lesions [23]. Other narrative reviews proposed the adoption of simplified PI-RADS scoring or the reservation of contrast medium for PI-RADS 3-4 lesions to offer improved management with fewer biopsies [24,25].

All these prior reviews mention study heterogeneity as a limitation but a more detailed analysis of what is missing and what should be included is still needed. Additionally, the documented dependency of the use of DCE on experience and MRI quality is under-reported and under-investigated in the reviews discussed above. This observation also matches emerging proposals for quality metrics in the prostate cancer diagnostic pathway, such as explicitly reporting information on each PI-RADS category in addition to explicitly commenting on image quality using the Prostate Imaging Quality (PI-QUAL) system [26,27,28]. In response to these observations, this review sought to understand and describe the extent of the study heterogeneity and the reporting on quality metrics by looking at the latest original research (within the past 5 years), published after the role of DCE was updated in 2015, that sought to answer the primary research question: *Can bpMRI replace mpMRI,* via *the omission of DCE, in the screening and assessment of clinically significant prostate cancer without diminishing diagnostic sensitivity/accuracy?* Additionally, this review sought to identify the key indications of DCE as reported in the PI-RADS v2.1 guidelines and assess the frequency with which these key indications and emerging quality metrics are reported in studies trying to answer the primary research question.

## 2. Methods

### 2.1. Paper Eligibility and Selection

**Figure 1:**
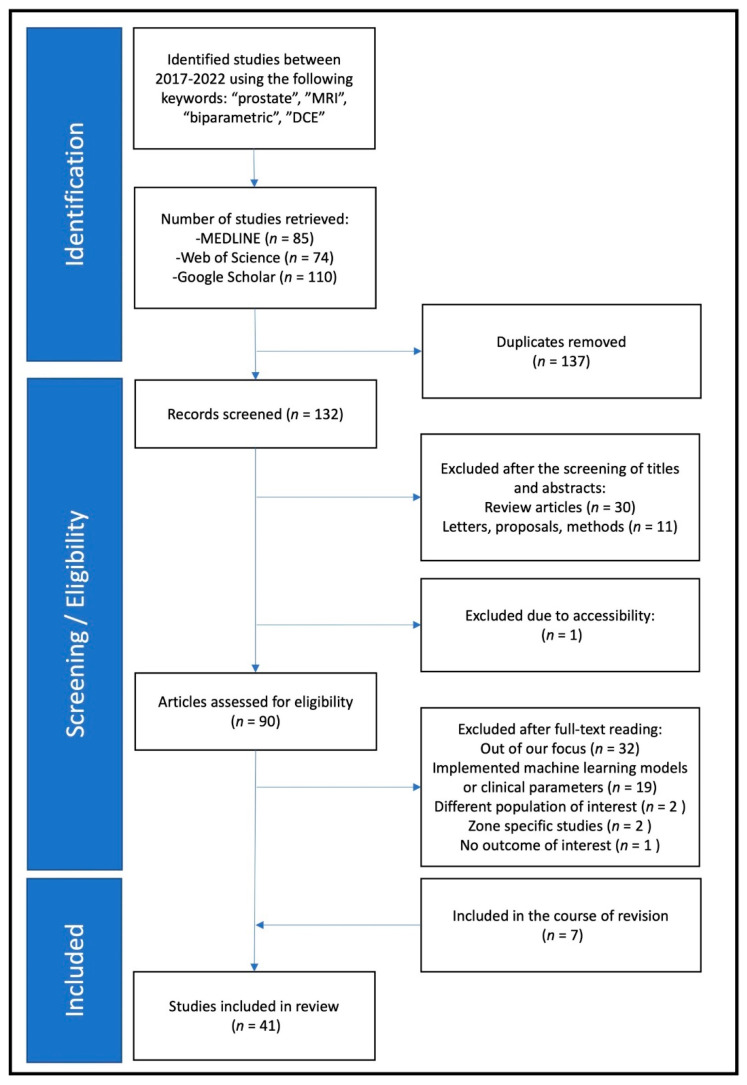
Study flow chart.

The key search terms used in medical databases to find eligible papers included the following: “prostate”, “MRI”, “biparametric”, and “DCE”. MEDLINE, Web of Science, Google Scholar, and PubMed were used to search for eligible papers published between January 2017 and April 2022. The inclusion criteria for papers included being an original manuscript, publication in the last five years, focus on bpMRI or mpMRI, and matching key words. All papers underwent title, abstract, and full-text screening. Papers were excluded if they had a different focus, were written in a language other than English, if incorrect outcome measures were reported, or if the paper was a review article or letter (Figure 1). Three reviewers independently searched for and screened all records.

### 2.2. Data Collection

Data from each study were reviewed and recorded in an Excel spreadsheet (Microsoft, Seattle, WA, USA). All information was captured either from full-text reading or by referencing Supplementary Materials if provided. In addition to general study details (authors, year of publication), the following were recorded: study design (prospective or retrospective), diagnostic test used in the study (i.e., bpMRI, mpMRI, clinical parameters, biomarkers), sample size (i.e., total number of patients, number of prostate cancer patients, and number of clinically significant prostate cancer patients), characteristics of the sample (i.e., age, Prostate Specific Antigen (PSA), PSA density, prostate volume), definition of clinically significant cancer according to the authors in the study, number of radiologists/trainees and their collective experience in the field in years, type of scoring system used by the readers (i.e., PI-RADS, Likert), the number of PI-RADS 3 lesions detected in bpMRI and/or mpMRI, preference of reference standard (biopsy or prostatectomy), exclusion of MRI images of low quality, and MRI technique (i.e., 3T or 1.5T MRI scanner, endorectal coil usage). In cases where data could not be obtained due to limitations in reporting, the value “did not indicate” (DNI) was assigned. Data extraction was conducted by three readers independently. Due to high variability in the studies, readers consulted with each other during the process regularly and came to an agreement in cases of discrepancies. All data were processed using Python and the following packages: matplotlib, pandas, numpy, scipy, and statsmodels. The method used to tabulate and visually display results was the plot function within the pandas module.

## 3. Results

A total of 132 unique records were identified by searching for the keywords in Google Scholar, MEDLINE, and Web of Science. These records then went through a screening process examining the titles and abstracts, after which 42 studies were excluded for the following reasons: *n* = 30 review articles, *n* = 11 letters/proposals/methods papers, *n* = 1 lack of accessibility. For the 92 remaining studies, full-text screening filtered out an additional *n* = 56 studies for the following reasons: *n* = 32 beyond the focus of this analysis, *n* = 19 combined bpMRI with machine-learning model or extra clinical variables, *n* = 2 different population of interest, *n* = 2 zone-specific studies, *n* = 1 no outcome of interest. Moreover, *n* = 7 additional studies were included during the revision. After all studies were fully screened, 41 studies remained for the final analysis.

### 3.1. Clinical Characteristics

Table 1 presents the clinical characteristics of the studies included in this review. Table 2 contains a summary of the papers that reported on each sub-population relevant to the topic. The following represent the percentages of papers reporting all sample sizes and sub-sample sizes. Out of the included studies, 100% (41/41) reported the total patient population, 80% (33/41) reported the proportion of any type of PCa in their population, 85% (35/41) reported the proportion of csPCa in their population, 27% (11/41) reported the proportion of PZ patients, 71% (29/41) reported the number of PI-RADS 3 lesion patients, and only 51% (21/41) reported csPCa PI-RADS 3 lesions.

### 3.2. Technical Characteristics

**Figure 2:**
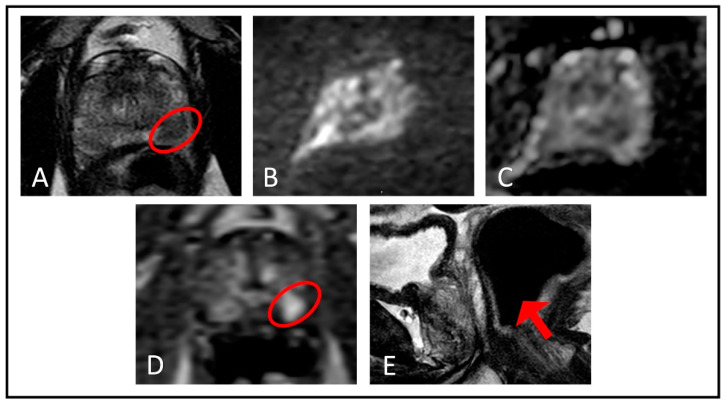
An example case from our own database demonstrating the added value of DCE MRI in the diagnosis of prostate cancer when DWI/ADC images are of insufficient quality, which may arise due to different reasons; in this case it was due to rectal gas-related distortion. (**A**) Axial T2-weighted imaging shows a hypointense lesion (red ellipse) in the left apical–mid peripheral zone. Both the (**B**) DWI and (**C**) ADC images are distorted due to rectal gas-related artifacts and unable to provide information regarding the hypointense lesion located in the left apical–mid peripheral zone. (**D**) Lesion demonstrates early focal enhancement (red ellipse) in dynamic contrast-enhanced imaging. (**E**) Rectal gas causing undiagnostic images in DWI and ADC sequences is depicted in the sagittal T2-weighted MRI (red arrow).

Table 3 presents the technical characteristics of the studies included in this review. Of the included studies (*n* = 41), 12 were prospective and 29 were retrospective analyses. Regarding the study cohorts, most were biopsy-naive (*n* = 26), two were repeat biopsy patients, five were patients with proven cancer, three had mixed populations, and five did not indicate the biopsy status of the patients. Nearly 46% (19/41) of the studies excluded MRI scans with insufficient quality due to artifacts caused by motion or rectal gas (Figure 2). The studies varied in their focus, with 32 providing head-to-head comparisons of bpMRI and mpMRI, 7 comparing bpMRI to historic mpMRI standards, and 2 focusing purely on mpMRI.

With respect to the definition of csPCa, 4 studies did not indicate one, 31 used the Gleason score (GS) ≥ 7 (3 + 4), and the remaining 6 used permutations of GS cutoffs ranging from 6–7 combined with the presence of an extra-prostatic extension (EPE), index lesion volume > 0.5 mL, max cancer core lengths (CCLs) ranging from 4–6 mm and Gleason grade group (GG) cutoffs ranging from 2–3. In terms of reference standard methods, 23 studies used biopsies, 6 preferred radical prostatectomies, and 10 utilized both procedures to confirm the presence of clinically significant cancers, with 2 studies not reporting the method of confirmation. With respect to MRI strength, 28 studies used 3T, 8 used 1.5T, and 4 used both 1.5T and 3T; 1 did not indicate. With respect to endorectal coil (ERC) use, only 5 studies used them while the remaining 36 omitted them from examinations. Across all studies, the median number of cumulative years of experience per study was 17 years (Range: 5–45) and the median number of readers per study was 2 (Range: 1–13). Variations in clinical parameters and technical characteristics are collectively presented in Table 4.

## 4. Discussion

This review analyzed papers from the last 5 years that had the goal of answering the following research question: *Can bpMRI replace mpMRI,* via *the omission of DCE, in the screening and assessment of clinically significant prostate cancer without diminishing diagnostic sensitivity/accuracy?* According to PI-RADS v2.1, the role of the DCE sequence in the staging of prostate cancer is primarily to assist in the appropriate grading of PI-RADS 3 lesions in the peripheral zone. Lesions that display early enhancement from DCE are upgraded to PI-RADS 4, potentially changing the course of patient treatment and care. It was also mentioned in PI-RADS v2.1 that when T2WI and DWI are of insufficient diagnostic quality, DCE plays a larger role in determining the PI-RADS assessment category. Therefore, any study trying to answer this research question should test for the experimental conditions for which DCE and thus I.V. contrast are indicated. The PI-RADS v2.1 indications for DCE are: (1) determining PI-RADS 3 lesions that include clinically significant prostate cancer; (2) assisting in the readout of MRIs with suboptimal diagnostic quality for T2WI and DWI sequences; and (3) assisting radiologists with relatively low experience in reading prostate MRIs. These indications help to assess the claims that DCE is a safety net for when T2WI/DWI sequences are unhelpful and to evaluate the reader experience-dependency of DCE, which has been demonstrated in the literature. These indications are also emerging as proposed quality metrics for prostate MRI that specifically endorse explicitly reporting information on each PI-RADS category in addition to explicitly commenting on image quality utilizing the Prostate Imaging Quality (PI-QUAL) system [26,27,28].

### 4.1. Studies Meeting the Majority of the Defined DCE Indications

The studies discussed in detail below are those that comprehensively reported PCa and csPCa rates within their equivocal lesion populations and either (1) also included multiple readers or (2) utilized 1.5T or did not explicitly mention removing bad quality scans. None of the studies met all the DCE indications defined previously.

Bao et al. [30] carried out a two-center, retrospective study of 638 individuals with the primary aim of comparing the diagnostic accuracy of bpMRI, mpMRI, and what they defined as optimized (Op) MRI, a combination of bpMRI and mpMRI based on PI-RADS 3 lesions. Their study utilized 3T MRI and included six radiologists (all with ≥1000 prostate MRI scans read), with two reading bpMRI (10 and 3 years of experience) according to PI-RADS v2.1 and two reading mpMRI (12 and 5 years of experience) using DCE. PI-RADS 3 lesions were assigned in 18.2% of cases (116/638) for bpMRI and 11.3% (72/638) for mpMRI. Using PI-RADS 3 as the diagnostic criterion, both methods had similar clinically significant cancer detection rates for both junior and senior radiologists. In this study, the interrater agreement between junior and senior readers was high and the interrater agreement between bpMRI and mpMRI was high. This study very elegantly showed the results of different protocols on the classification of equivocal lesions and provided evidence that junior readers may perform similarly to seniors with and without DCE. It is worth noting that this study specifically excluded MRI scans with poor image quality and imaging exams from outside institutions.

Bosaily et al. [33] carried out an extension of the PROMIS study, which is a large, multi-center, prospective study of 497 biopsy-naive individuals with the primary aim of assessing the diagnostic accuracy of pre-biopsy mpMRI using standard 1.5T machines without endorectal coil. This study extension was not statistically powered to evaluate differences in sequences. The authors found that the addition of DCE was helpful in correctly identifying any csPCa (GS ≥ 7 (3 + 4) irrespective of cancer core length) lesions compared to both T2WI and T2WI + DWI. The addition of DCE reduced the number of equivocal scores (3/5) slightly, with 28% of patients classified as equivocal compared with 32% using T2WI + DWI alone. Their findings did not show a meaningful difference between bpMRI and mpMRI depending on the definition of clinically significant prostate cancer, with other considered definitions including cancer core (GS ≥ 7 (3 + 4) or cancer core length ≥ 4) or without cancer core (GS ≥ 7 (4 + 3) irrespective of cancer core). However, their overall conclusions mentioned that the addition of DCE did not significantly improve the diagnostic accuracy of T2WI + DWI. This study shows some of the variation that arises from different definitions of clinically significant prostate cancer and how the conclusions on the utility of DCE depend on a thorough understanding and evaluation of equivocal lesions. This study did not evaluate the dependence of reader experience or scan quality, but their detailed analysis of equivocal lesions is invaluable.

Cereser et al. [37] carried out a retrospective study on a prospectively collected database of 108 individuals with the primary aim of comparing multiple mpMRI-derived protocols in detecting csPCa. This study incorporated two readers with varying experience levels (R1 600 cases vs. R2 250 cases) and evaluated each imaging sequence in series with DCE being the last addition. The strengths of this study include its histopathological mapping of whole-mount histopathology sections to MRI and comprehensive reporting on their equivocal lesion population. The interrater agreement was found to be highest for mpMRI compared to bpMRI; however, these protocols showed comparable cancer detection rates with no significant interrater differences considering a threshold of PI-RADS ≥ 3. DCE influenced the final PI-RADS score in 8% (11/137) of observations for R1 and 7.7% (9/117) of observations for R2. The csPCa rates in the upgraded category 3 to category 4 lesions for each reader were 54.5% (6/11) for R1 and 88.9% (8/9) for R2. Their overall conclusions were that including DCE imaging has the potential to minimize PI-RADS v2 category 3 observations while prompting appropriate biopsies.

Brancato et al. [34] carried out a retrospective study of 111 patients with 117 lesions with the primary aim of measuring the added value of DCE-MRI in combination with T2WI + DWI with respect to both reproducibility and diagnostic accuracy. This study included three separate radiologists all with similar years of experience (7–10 years). They found that the best overall results for interrater agreement were reached when considering only csPCa (GS ≥ 7 (3 + 4) irrespective of cancer core length) and an mpMRI-based PI-RADS classification. However, the overall findings related to diagnostic accuracy revealed that the PI-RADS scoring in bpMRI protocols was comparable to that assigned in the mpMRI protocol. This study did not perform a sub-analysis for equivocal lesions or mention the quality of the scans included.

Han et al. [46] carried out a retrospective study of 123 individuals with the primary aim of comparing the performance of bpMRI and mpMRI combined with PSAD in detecting clinically significant prostate cancer. This study only included patients with a PSA between 4–10 ng/mL. For image analysis, this study included two separate radiologists both with >5 years of experience, and both radiologists first read the scans as bpMRI, took one-month washout, and then read the scans again as mpMRI (including DCE). This study comprehensively analyzed the detection rates for each PI-RADS category and found that in approximately 10.6% (13/123) of their population, DCE influenced the final PI-RADS score. Looking at their PI-RADS category 3-specific findings, there were eight lesions upgraded to category 4 and 62.5% (5/8) of these DCE-positive lesions were upgraded from the added DCE findings, which were from prostatitis. There were some discrepancies between category 3 and 4 lesion assignments in the csPCa detection rates for category 3 lesions (mpMRI: 3/7 csPCa, bpMRI: 4/18 csPCa) and category 4 lesions (mpMRI: 16/29, bpMRI: 15/21). Although there were multiple readers, this study did not assess the level of agreement or individual assignment mismatch. This study also did not mention the quality of the MRI scans or removal of low-quality scans. The overall results from this study suggest bpMRI achieves better performance than mpMRI in detecting csPCa, considering a significance threshold of PI-RADS ≥ 3.

Junker [48] et al. carried out a retrospective study of 236 patients with the primary aim of investigating if and how omitting DCE influences diagnostic accuracy and tumor detection rates. This study utilized 1.5T and 3T scanners with endorectal coil. Image interpretation was carried out by one experienced uro-radiologist who first retrospectively reviewed MRI datasets without DCE using PI-RADS v2, then conducted an evaluation with DCE in the exact same reading session. DCE influenced the final PI-RADS v2 score in 9.75% (23/236) patients. There were a total of 135 PCa lesions and utilizing bpMRI led to the downgrading of 5.93% of PCa lesions (8/235) from PI-RADS category 4 to category 3 and 62.5% (5/8) of these PCa lesions were GS = 7 (3 + 4), or clinically significant cancer. This study also defined another cutoff for clinically significant cancer (GS ≥ 7 (4 + 3)), for which they found no significant differences between bpMRI and mpMRI, largely due to the exclusion of PCa lesions, primarily pattern 3. No PCa lesions were downgraded from higher scores to a score < 3; therefore, no additional PCa was scored as benign or completely missed. Limitations related to this study involved the interpretation of bpMRI and mpMRI in the same session, and there was no sub-analysis or mention of scan quality. The overall results from this study suggest that omitting DCE did not lead to significance differences in the diagnostic accuracy of tumor detection rates; however, this was with respect to a clinically significant cancer definition of GS ≥ 7 (4 + 3).

Pesapane et al. [54] carried out a retrospective analysis of 431 individuals with the primary aim of comparing the performance of mpMRI and bpMRI in PCa detection in individuals with elevated PSA levels. This study utilized a 1.5T scanner and ERC, excluding patient scans with artifacts and scans without ERC. This study included two radiologists with different amounts of experience (3 years vs. 5 years) in the interpretation of prostate MRIs. bpMRI readouts were performed first and then, after a one-month washout period, mpMRI readouts with DCE were conducted. Intrareader agreement was found to be substantial. DCE influenced the PI-RADS score of 6% (25/431) of scans for reader 1 and 8% (35/431) for reader 2. For high-grade PCa cases, for bpMRI, R1 and R2 had a sensitivity of 84% and 80%, and the specificity was 77% and 74% for R1 and R2, respectively. For mpMRI, the sensitivity was 86% and 80% and specificity was 78% and 74% for R1 and R2, respectively. The overall results from this study suggest there is no significant reduction in diagnostic performance of bpMRI compared to mpMRI. This study did not perform a sub-analysis and explicitly described removing scans with artifacts and those without endorectal coil.

Tamada [59] et al. carried out a retrospective analysis of 103 patients (with 165 suspected PCa lesions) with the primary aim of comparing the interobserver reliability and diagnostic performance of bpMRI compared to mpMRI using PI-RADS v2.1. This study utilized a 3T scanner with a pelvic phased-array coil and included three radiologists with widely ranging experience levels (8 years R1, 12 years R3, and 22 years R2). The bpMRI and mpMRI reading sessions were conducted in the same session with the traditional flow of bpMRI first and mpMRI second. The interrater reliability was shown to have good agreement for both bpMRI and mpMRI but was lowest for PZ lesions. This study had two separate definitions of clinically significant cancer: (1) tumor with GS ≥ 7 and tumor diameter ≥ 5 mm or (2) tumor with GS = 3 + 3 and tumor size ≥ 0.5 mL (tumor diameter ≥ 8 mm). When comparing the diagnostic performance for csPCa detection between bpMRI and mpMRI, they found that diagnostic sensitivity was significantly higher in all readers for mpMRI, but diagnostic specificity was significantly lower in all readers compared to bpMRI. For their PI-RADS categories 3 and 4-specific sub-analysis, the false-positive rate for upgrading PI-RADS category 3 to category 3 + 1 (category 4) for the readers was 62% R1 (12/21), 73% R2 (19/26), and 57% R3 (12/21). The high false-positive rate may have resulted from the enhancement effect on DCE-MRI in benign prostatic conditions such as prostatitis and fibrosis. The overall conclusion for this study suggests that bpMRI may be acceptable for detecting csPCa; however, solutions need to be sought to improve diagnostic sensitivity. This study did not perform a sub-analysis based on scan quality nor mention any effects of quality on interpretation.

The studies above represent those that comprehensively reported on their PI-RADS category 3 lesion population and they give many valuable insights into the potential utility of DCE and its limitations. For the studies with multiple readers that reported on their csPCa PR3 population, many of these studies showed good interrater agreement between junior and senior readers, with the highest interrater agreements generally being ascribed to mpMRI. The impact of removing DCE on diagnostic accuracy in the studies above largely depended on their definition of clinically significant prostate cancer, with studies using a definition of GS ≥ 7 (3 + 4) tending to show superiority for mpMRI, in contrast to studies that used other definitions (GS ≥ 7 (4 + 3), which tended to show equivalent performance. The impact of DCE was also heavily dependent on the significance threshold for biopsies, which in many studies was identified to be PI-RADS category ≥ 3. Due to the comprehensive reporting on their PI-RADS equivocal populations, it is easy to understand how changing this significance threshold would significantly impact results. It is also noteworthy that this significance threshold for biopsies was not uniformly agreed on and is another area of controversy and active research. All studies that comprehensively reported on their PI-RADS equivocal populations found that DCE was required for the final PI-RADS assessment in approximately 6–10% of their cohorts due to upgrading from category 3 to category 4. Additionally, all these studies either did not mention scan quality as a variable or they explicitly removed MRI scans with low quality. This last point emphasizes that the current results and conclusions regarding the utility of DCE must be understood within the special context in which all images are of higher diagnostic quality. However, as mentioned previously, in the context of decreased scan quality, DCE has shown evidence of having more robust quality compared to DWI or ADC maps [10,16,17].

### 4.2. DCE Indications and Frequency of Reporting

Although 100% (41/41) of the included papers sought to answer the primary research question, only 71% (29/41) of the included papers reported the number of PI-RADS 3 lesions in their respective patient cohorts. An even smaller percentage (51%, 21/41) reported what proportion of these PI-RADS 3 lesions included clinically significant prostate cancer. Thus, most of the papers that were trying to answer the primary research question did not report key elements of indication #1 and, as a result, their findings are much harder to interpret for the population of interest. Indication #1 is extremely important to report since the prevalence of PI-RADS 3 lesions and of csPCA within these lesions is hospital/institution-dependent. The papers described below are those in the minority that reported their PI-RADS 3 population. The results of one of the included studies [7] emphasize the importance of understanding the population, as they provide tumor burden-dependent suggestions. They suggest bpMRI for patients with average risk and PSA < 10 ng/mL for whom EPE is minimal but the risk of cancer mortality is not marginal. They then suggest that patients with higher PSA, among whom EPE is more common, use the full mpMRI examination for assessment of cancer. These disease burden-dependent suggestions rely heavily on understanding and reporting the population under study, and if studies attempting to answer the primary research question do not address this transparently, they will be severely limited. Another study [49] that was in the minority of papers that reported their PI-RADS 3 population investigated bpMRI and concluded that PI-RADS 3 lesions are expected to require additional tools to supplement bpMRI based on their results as compared to other PI-RADS lesions. This emphasizes the importance of reporting PI-RADS 3 prevalence in a study to enable more accurate research conclusions. Another study [36] that reported the PI-RADS 3 population in detail found that when the high-risk threshold was low (0.1 to 0.45), bpMRI was superior to mpMRI. However, when the risk interval was larger than 0.5, PI-RADS v2 with incorporation of DCE was better than bpMRI. This paper found that for lesions that were in category 3 in bpMRI, the application of DCE via PI-RADSv2 improved cancer detection. This study provides further support for the importance of reporting the PI-RADS 3 lesion population and thus highlights the limits of papers making conclusions about the utility of DCE without talking at all about PI-RADS 3 lesions.

Further, most (68.3%, 28/41) of the included papers used high-quality 3.0 Tesla scanners, minimizing the possibility of low-quality T2WI and DWI sequences. With regard to image quality, 46% (19/41) of studies made explicit statements about removing low-quality MRI patient scans resulting from MRI noise, motion, or artifacts. These key areas of low-image quality are where DCE has been indicated to be a strength. On assessing the utility of DCE for patient cases where the T2WI and DWI sequences are low quality (indication #2), 0% (0/41) of the included papers included any details about their MRI specifics or performed any type of sub-analysis or result stratification based on image sequence quality. If studies only include scans with high-quality T2WI and exclude scans with motion/artifacts/noise that make the T2WI and DWI non-diagnostic, then these studies will not accurately reflect real-world scenarios. Thus, every included paper trying to answer the primary research question omitted analyzing indication #2 and thus their findings are much harder to interpret for the population of interest.

Finally, only a small number of the studies included head-to-head comparisons of multiple readers with varying experience. Prior research has found that experienced readers can maintain high diagnostic accuracy when reading bpMRIs while less experienced readers strongly benefit from the DCE sequence [44,65]. The accuracy of detection of prostate cancer is higher for less experienced readers when DCE is included [65]. Thus, most of the included studies trying to answer the primary research question additionally omitted analyzing indication #3 and thus their findings are much harder to interpret for the population of interest.

In summary, without complete and transparent reporting of information concerning indications #1–3 of DCE, it is impossible to stratify studies, pool results in meta-analyses, and confidently answer whether bpMRI can replace mpMRI via the omission of DCE. Future meta-analyses and original studies are encouraged to analyze data and include papers that address indications 1–3 for DCE.

### 4.3. Study Heterogeneity

It is a challenging task to compare these diagnostic studies solely based on the statistical metrics as most studies used different definitions of clinically significant cancer. Moreover, since there is a lack of a uniform approach before deciding on a biopsy, it would be impractical to comment on the diagnostic performance of the aforementioned methods. However, it could be concluded from the vast ranges of the metrics that variation is huge, which might be explained by the difference in the experience of the readers and the diversity of the biopsy and clinically significant cancer criteria adopted among the studies. It should also be kept in mind that some of the cancers could have gone unnoticed by the studies that used biopsies (*n* = 23) as a reference standard method rather than radical prostatectomies. Therefore, we can conclude that high variability exists in both diagnostic methods in terms of sensitivity, specificity, and AUC value. Implementing more standardized approaches in the future might make it possible to compare the diagnostic performance of bpMRI and mpMRI.

### 4.4. Limitations

Our study has four main limitations. First, using a 5-year cutoff as an inclusion criterion prevented the addition of older studies who might have answered the primary research question better through more transparent reporting. However, the concept of bpMRI was introduced into the prostate MRI research world relatively recently and the roles/responsibilities of the mpMRI sequences have changed over the years. Thus, including studies using PI-RADS v1 would have been a limitation. Second, because of the inclusion and exclusion criteria, the sample size for the studies was relatively small (*n* = 41). Third, many of the studies did not explicitly state the proportion of PI-RADS 3 patients, which was instead calculated by percentages given in the tables or within the text. This limitation was also pertinent when searching for the proportion of the PI-RADS 3 patients that had PCa or csPCa. Fourth, there was also the possibility of overlap between the patient cohorts of the included studies, but our aim was to demonstrate the heterogeneity of the papers matching our inclusion criteria.

### 4.5. Conclusions

This review sought to more thoroughly investigate study heterogeneity and reporting regarding primary DCE indications in studies trying to answer the primary research question: *Can* *bpMRI replace mpMRI,* via *the omission of DCE, in the screening and assessment of clinically significant prostate cancer without diminishing diagnostic sensitivity/accuracy?* The PI-RADS v2.1 indications for DCE are: (1) determining PI-RADS 3 lesions that include clinically significant prostate cancer; (2) assisting readout of MRIs with suboptimal diagnostic quality for T2WI and DWI sequences; and (3) assisting radiologists with relatively low experience in reading prostate MRIs. These indications have been endorsed as emerging quality metrics for prostate MRI and are especially important when discussing the utility of DCE [26,27,28]. In summary, most papers that try to answer the primary research question omit results/discussions relating to indications 1–3 and as a result their conclusions and interpretations, taken both independently and combined in systematic reviews, are weakened. For the studies that fulfilled most of the DCE indications 1–3, it can be concluded that the utility of DCE is very definition-dependent. The definition of csPCa as GS ≥ 7 (3 + 4) and a cancer significance threshold of PI-RADS > 3 tend to show the benefit of DCE, as compared to other definitions of csPCa or thresholds. Additionally, none of these studies mentioned including scans of low quality, with some explicitly excluding low-quality MRIs, which is a potential area of strength for DCE. Thus, the conclusions regarding the utility of DCE in most published studies should be taken with special consideration of the population under study, with current evidence supporting its equivalent use specifically for images of all-good diagnostic quality. Prospective evaluation that incorporates and reports on these indications and emerging quality metrics is needed to truly answer the question under debate. It is the authors’ view that future reviews and studies aiming to answer the primary research question should transparently report all data related to indications 1–3 for the most complete and accurate conclusions regarding the utility of DCE. Moreover, there is a need for a standardized reporting system designed for bpMRI; without it, such heterogeneity is likely to persist in future studies.

## Figures and Tables

**Table 1:** Clinical parameters. * = et al., ** = Likert, (R1, R2), commas between numbers indicate output from multiple readers.

Author (Citation)	Year	Number of Patients								Age in Years (Mean/Median)	PSA in ng/mL (mean/median)	Prostate Volume in mL	PSA Density in ng/mL^2^
		Total	PCa	csPCa	PZ Lesion	PR-3/LK-3 Lesions							
						bpMRI		mpMRI					
						Overall	csPCa	Overall	csPCa				
Al Salmi * [29]	2020	100	35	28	DNI	5, 5	DNI	6, 1	DNI	64	10.3	69.9	0.17
Bao * [30]	2021	638	319	287	338	116	45	72	16	69	DNI	DNI	DNI
Barth * [31]	2017	63	60	28	DNI	DNI	DNI	DNI	DNI	62.2	9.2	DNI	DNI
Boesen * [32]	2018	1020	655	404	DNI	130	17	DNI	DNI	67	8	53	0.15
Bosaily * [33]	2020	497	293	61	DNI	158	37	136	27	64	6.5	DNI	DNI
Brancato * [34]	2020	111	72	38	105	43, 28, 35	12, 8, 8	17, 9, 15	3, 3, 4	69	DNI	57.5	0.26
Brembilla * [35]	2022	151	129	76	DNI	20	DNI	21	DNI	62	6.8	DNI	DNI
Cai * [36]	2021	224	90	85	77	82, 85	4	53, 58	1, 2	69	14.55	DNI	DNI
Cereser * [37]	2020	108	104	47	DNI	24, 12	16, 11	13,3	10, 3	64.8	8.4	DNI	DNI
Cho * [38]	2020	41	DNI	41	DNI	DNI	DNI	1	DNI	64.3	9.2	33.1	0.31
Choi * [39]	2019	113	113	84	DNI	23, 35	15, 21	10, 13	6, 7	65	7.9	DNI	DNI
Christophe * [40]	2020	92	DNI	DNI	DNI	DNI	DNI	DNI	DNI	63	DNI	DNI	0.24
Di Campli * [41]	2018	85	72	41	DNI	DNI	DNI	DNI	DNI	70,39	8.5	DNI	DNI
EL-Adalany * [42]	2021	60	35	33	DNI	17	12	7	2	65	35	DNI	DNI
Eldred-Evans * [43]	2020	246	209	103	DNI	81 **	DNI	83 **	DNI	62	6.8	37	DNI
Gatti * [44]	2019	65	DNI	45	43	DNI	DNI	DNI	DNI	65 (cases), 62 (controls)	7.5 (cases), 6.35 (controls)	61.3 (cases), 47.7 (controls)	0.11(cases), 0.125 (controls)
Giannarini * [45]	2021	108	34	74	DNI	DNI	DNI	DNI	DNI	64.8	8.4	DNI	DNI
Han * [46]	2020	123	50	37	13	18	4	10	3	66.3	7.227	DNI	0.207
Jambor * [47]	2019	338	207	146	DNI	66 **	8 **	DNI	DNI	64	6.9	39	0.17
Junker * [48]	2019	236	135	DNI	DNI	69	20	48	12	67.6	6.4	45	DNI
Kim * [49]	2019	140	66	37	DNI	57	11	DNI	DNI	67.2	8.1	49	0.16
Knaapila * [50]	2021	639	410	307	DNI	110 **	13 **	DNI	DNI	54	8.9	43	0.23
Kobilnyk * [51]	2020	26	14	DNI	23	8	4	DNI	DNI	67.6	DNI	DNI	DNI
Kuhl * [52]	2017	542	180	139	DNI	DNI	DNI	DNI	DNI	64.8	8.5	57.4	0.15
Lee * [53]	2017	123	35	9	DNI	DNI	DNI	DNI	DNI	61.8–62	6.19–6.7	38.6–40.2	0.17–0.20
Obmann * [6]	2018	129	84	45	DNI	49	11	DNI	DNI	61.8	8.04	53.6	0.15
Pesapane * [54]	2021	431	195	65	DNI	119, 132	DNI	95, 100	DNI	61.5	12	58	0.18
Roh * [55]	2020	594	DNI	DNI	332	69	10	DNI	DNI	66	7.6	60	0.17
Russo * [7]	2021	311	117	94	DNI	26	DNI	9	DNI	66.3	5.68	49.6	0.17
Scialpi * [56]	2017	41	41	22	22	DNI	DNI	DNI	DNI	64.5	7.8	DNI	DNI
Sherrer * [57]	2019	344	DNI	DNI	DNI	DNI	DNI	DNI	DNI	65	7.61	DNI	DNI
Taghipour * [58]	2019	271	271	212	209	24	8	DNI	DNI	59	6.7	DNI	DNI
Tamada * [59]	2021	103	DNI	81	78	73, 57, 58	39, 36, 32	34, 21, 26	DNI	69.8	6.92	DNI	DNI
Thestrup * [60]	2019	101	101	27	DNI	23	DNI	21	DNI	64	6.3	49	0.13
van der Leest * [18]	2019	626	334	190	DNI	49	11	40	9	65	6.4	56	0.11
De Visschere * [61]	2017	245	DNI	144	DNI	20	8	DNI	DNI	66	9	49.3	DNI
Wallstrom * [62]	2021	551	DNI	DNI	DNI	59	DNI	33	DNI	57	3.3	41	0.075
Wang * [63]	2020	109	28	15	109	DNI	39	DNI	DNI	65–69	8.11–15.52	35.31–53.48	0.20–0.48
Wang * [64]	2021	224	79	18	DNI	86	DNI	DNI	DNI	65–71	9.84–37.24	DNI	DNI
Xu * [12]	2019	235	122	99	DNI	29	DNI	16	DNI	66.87	4.65	DNI	DNI
Zawaideh et al. [16]	2020	264	171	93	DNI	DNI	DNI	DNI	DNI	65	6.08	50.55	0.11

PCa: prostate cancer, csPCa: clinically significant prostate cancer, PZ: peripheral zone, PR-3: PI-RADS category 3, bpMRI: biparametric MRI, mpMRI: multiparametric MRI, PSA: prostate-specific antigen, DNI: did not indicate.

**Table 2:** Clinical parameter population summarized.

	Total Patients	PCa Patients	csPCa Patients	PZ Patients	PR3 bpMRI	csPCa PR3 bpMRI	PR3 mpMRI	csPCa PR3 mpMRI
Max	1020	655	404	338	158	45	136	27
Min	26	14	9	13	8	4	1	2
Median	151	104	65	78	49	11	21	10.5
Total	10,468	4860	3255	1349	1027	241	414	69
Papers Reporting	41	33	35	11	29	21	20	10
Proportion Reporting	1	0.8	0.85	0.27	0.71	0.51	0.49	0.24

PCa: prostate cancer, csPCa: clinically significant prostate cancer, PZ: peripheral zone, PR-3: PI-RADS category 3, bpMRI: biparametric MRI, mpMRI: multiparametric MRI.

**Table 3:** Technical parameters.

Author (Citation)	Year	Study Type	Study Cohort	Excluded Pts. with Bad Quality MRI	Diagnostic Test	Definition of csPCa	MRI Findings Considered Positive/Bx Thresholds	Bx or RP	Field Strength	ERC Usage	No. of Readers	Total Experience (Years)
Al Salmi * [29]	2020	Pro	DNI Bx status	Y	Both	GS ≥ 7 (3 + 4)	DNI	DNI	3T	N	2	DNI
Bao * [30]	2021	Ret	DNI Bx status	Y	Both	GS ≥ 7 (3 + 4)	PR ≥ 3	DNI	3T	N	4	30
Barth * [31]	2017	Pro	Bx-naive	Y	Both	GS ≥ 7 (3 + 4)	All	Bx	3T	Y	3	29
Boesen * [32]	2018	Pro	Bx-naive	N	bpMRI	GS ≥ 7 (4 + 3), CCL > 50% for GS 7 (3 + 4)	PR ≥ 3 + PR_all	Both	3T	N	1	5
Bosaily * [33]	2020	Pro	Bx-naive	N	Both	GS ≥ 7(3 + 4), ≥4 mm CCL	LK ≥ 2	Bx	1.5T	N	DNI	DNI
Brancato * [34]	2020	Ret	Bx-naive	N	mpMRI	GS ≥ 7 (3 + 4)	All	Bx	1.5T	Y	3	25
Brembilla * [35]	2022	Ret	DNI Bx status	N	Both	GS ≥ 7 (3 + 4)	All	Bx	3T	N	3	28
Cai * [36]	2021	Ret	Bx-naive	N	bpMRI	GS ≥ 7 (3 + 4)	DNI	Bx	1.5T	N	2	5
Cereser * [37]	2020	Ret	Proven Ca	Y	Both	GS ≥ 7 (3 + 4)	DNI	RP	3T	N	2	600 cases/250 cases
Cho * [38]	2020	Ret	Bx-naive	N	Both	GS ≥ 6, V > 0.5 mL	PR ≥ 4	RP	3T	N	2	18
Choi * [39]	2019	Ret	Proven Ca	N	Both	GS ≥ 7 (3 + 4), EPE, V > 0.5 mL	PR ≥ 3	Bx	3T	N	2	20
Christophe * [40]	2020	Ret	DNI Bx status	Y	Both	DNI	All	RP	3T	N	4	17
Di Campli * [41]	2018	Ret	Bx-naive	Y	Both	GS ≥ 7 (3 + 4), EPE	PR ≥ 3 (overall), PR ≥ 4 (csPCa)	Both	1.5T	N	3	11
EL-Adalany * [42]	2021	Pro	Bx-naive	Y	Both	GS ≥ 7 (3 + 4), EPE, V > 0.5 mL	All	Both	3T	N	2	19
Eldred-Evans * [43]	2020	Ret	Repeat Bx	Y	Both	GS ≥ 7 (3 + 4), ≥ 6 mm CCL of any GS	LK ≥ 3	Bx	3T	N	1	10
Gatti * [44]	2019	Ret	Bx-naive	N	Both	DNI	All	Both	1.5T	N	6	DNI
Giannarini * [45]	2021	Ret	Proven Ca	Y	Both	≥pT3	≥pT3	RP	3T	N	2	12
Han * [46]	2020	Ret	Bx-naive	Y	Both	GS ≥ 7 (3 + 4)	All	Bx	3T	N	2	10
Jambor * [47]	2019	Pro	Bx-naive	Y	bpMRI	GS ≥ 7 (3 + 4)	LK ≥ 3	Bx	1.5T, 3T	N	3	DNI
Junker * [48]	2019	Ret	Bx-naive	N	Both	GS ≥ 7 (4 + 3)	PR ≥ 3	Both	1.5T, 3T	N	1	DNI
Kim * [49]	2019	Ret	Bx-naive	Y	bpMRI	GS ≥ 7 (3 + 4)	PR ≥ 3	Bx	3T	N	2	19
Knaapila * [50]	2021	Pro	Mixed	N	bpMRI	GS ≥ 7 (3 + 4)	LK ≥ 3, LK ≥ 4	Both	1.5T, 3T	N	DNI	DNI
Kobilnyk * [51]	2020	Ret	Bx-naive	N	bpMRI	GS ≥ 7 (3 + 4)	PR ≥ 4	Bx	1.5T	N	DNI	DNI
Kuhl * [52]	2017	Ret	Repeat Bx	Y	Both	GS ≥ 7, PSA ≥ 20, stage > T2b-T3a	PR ≥ 3	Bx	3T	N	4	DNI
Lee * [53]	2017	Ret	Bx-naive	N	Both	GG > 3, >5 mm CCL	DNI	Both	3T	N	2	DNI
Obmann * [6]	2018	Pro	Bx-naive	Y	bpMRI	GS ≥ 7 (3 + 4)	PR ≥ 3	Bx	3T	N	1	14
Pesapane * [54]	2021	Ret	DNI Bx status	Y	Both	GS ≥ 7 (3 + 4), EPE, GG ≥ 7 (4 + 3)	PR ≥ 3	Both	1.5T	Y	2	8
Roh * [55]	2019	Ret	Bx-naive	N	mpMRI	GS ≥ 7 (3 + 4)	PR ≥ 3	Bx	3T	DNI	13	6–38
Russo * [7]	2021	Pro	Bx-naive	N	Both	GS ≥ 7 (3 + 4)	PR ≥ 3, PSAD ≥ 0.12	Bx	1.5T	Y	DNI	DNI
Scialpi * [56]	2017	Ret	Proven Ca	Y	Both	GS ≥ 7 (3 + 4)	All	RP	3T	N	2	DNI
Sherrer * [57]	2019	Ret	Mixed	N	Both	DNI	DNI	Bx	DNI	N	DNI	DNI
Taghipour * [58]	2018	Ret	Proven Ca	N	Both	GS ≥ 7 (3 + 4)	All	RP	3T	Y	1	14
Tamada * [59]	2021	Ret	Bx-naive	N	Both	GS > 6	All	Both	3T	N	3	42
Thestrup * [60]	2019	Pro	Bx-naive	N	Both	GS ≥ 7 (3 + 4)	PR ≥ 3	Bx	3T	N	1	6
van der Leest * [18]	2019	Pro	Bx-naive	N	Both	GS ≥ 7 (3 + 4)	PR ≥ 3	Bx	3T	N	2	30
De Visschere * [61]	2017	Ret	Bx-naive	N	Both	GS ≥ 7 (3 + 4)	All	Bx	3T	N	DNI	DNI
Wallstrom * [62]	2021	Pro	Bx-naive	Y	Both	DNI	PR ≥ 4	Bx	3T	N	3	22
Wang * [63]	2020	Ret	Bx-naive	N	Both	GS ≥ 7 (3 + 4)	PR ≥ 3	Bx	3T	N	2	10
Wang * [64]	2021	Ret	Bx-naive	Y	Both	GS ≥ 7 (3 + 4)	DNI	Bx	3T	N	2	16
Xu * [12]	2019	Ret	Bx-naive	Y	Both	GS ≥ 7 (3 + 4), EPE, V > 0.5 mL	All	Both	3T	N	2	18
Zawaideh * [16]	2020	Ret	Mixed	N	Both	GS ≥ 7 (3 + 4)	LK ≥ 3, LK ≥ 4	Bx	1.5T, 3T	N	1	DNI

Pro: prospective, Ret: retrospective, Y/N: yes/no, Bx: biopsy, csPCa: clinically significant prostate cancer, PR: PI-RADS, LK: Likert, PR-3: PI-RADS category 3, EPE: extraprostatic extension, V: lesion volume, CCL: cancer core length, GS: Gleason score, PSAD: PSA density, RP: radical prostatectomy, ERC: endorectal coil, DNI: did not indicate. * = et al.

**Table 4:** Summary of heterogeneity in the included studies.

**Study Type**	**Number of Studies**	**Definition of csPCa**	**Number of Studies**
Retrospective	29	GS ≥ 7 ± other parameters	31
Prospective	12	DNI	4
**Diagnostic Test**		Pathological stage ≥ pT3	1
Both	32	GG > 3, >5 mm CCL	1
bpMRI	7	GS ≥ 7 (4 + 3), CCL > 50% for GS 7 (3 + 4)	1
mpMRI	2	GS > 6	1
		GS > 7 (4 + 3)	1
**Study Cohort**		GS ≥ 6, V > 0.5 mL	1
Bx-naive	26	**Removed MRI with Artifacts**	
Mixed	3	Yes	19
Proven Ca	5	No	22
DNI Bx status	5		
Repeat Bx	2		
**Bx Thresholds and Number of Studies**			
**PIRADS**	**17**	**Likert**	**5**
PIRADS ≥ 3	11	Likert ≥ 3	2
PIRADS ≥ 4	3	Likert ≥ 3, Likert ≥ 4	2
PIRADS ≥ 3, PSAD ≥ 0.12	1	Likert ≥ 2	1
PIRADS ≥ 3 (overall), PIRADS ≥ 4 (csPCa)	1	≥pT3	1
PIRADS ≥ 3 + PIRADS all	1	DNI	6
		All	12
**Reference Standard Methods and Number of Studies**			
Bx	23	RP	6
Bx and RP	10	DNI	2
**Field Strength**	**Number of Studies**	**ERC**	**Number of Studies**
3T	28	Did not use	35
1.5T	8	Used	5
1.5T, 3T	4	DNI	1
DNI	1		
**No. of Readers**	**Number of Studies**	**Collective Experience of the Readers in Years**	**Number of Studies**
Median of 2 (Range 1–13)	35	Median of 17 (Range 5–42)	25
DNI	6	DNI	16

csPCa: clinically significant prostate cancer, EPE: extraprostatic extension, V: lesion volume, CCL: cancer core length, GS: Gleason score, PSAD: PSA density, Bx: biopsy, RP: radical prostatectomy, ERC: endorectal coil, DNI: did not indicate.

## Data Availability

Images courtesy of Molecular Imaging Branch, NCI.
